# German translation, cultural adaptation and linguistic validation of the PedsQL healthcare satisfaction module

**DOI:** 10.1186/s12955-026-02492-1

**Published:** 2026-02-13

**Authors:** Alexandra Piltz, Clara Herta Spiller, Anja Zschieschang, Guido Fitze, Holger Muehlan, Jurek Schultz

**Affiliations:** 1 Department of Pediatric Surgery, Carl Gustav Carus Medical Faculty, TUD Dresden University of Technology, Fetscherstraße 74, 01307 Dresden, Germany; 2 Division for Medical Psychology, Department of Medicine, HMU Health & Medical University Erfurt, Anger 64–73, 99084 Erfurt, Germany; 3 German Center for Child & Adolescent Health (DZKJ), Site Greifswald/Rostock, Germany; 4 Pediatric and Adolescent Surgery and Urology, Klinikum Chemnitz gGmbH, Flemmingstraße 2, 09116 Chemnitz, Germany

**Keywords:** Healthcare satisfaction, Patient-reported outcomes, PedsQL, Cultural adaptation, Linguistic validation, Qualitative content analysis

## Abstract

**Background:**

Healthcare satisfaction (HCS) is a crucial patient-reported outcome that is essential for evaluating medical care quality from the patient’s viewpoint and may also significantly impact other patient-reported outcome measures. However, currently validated German instruments lack a generic focus, which is essential for treating various diagnoses in pediatric surgery contexts. This study presents the translation and cultural adaptation of the PedsQL Healthcare Satisfaction Generic Module (HCSGM) for Germany.

**Methods:**

The PedsQL 3.0 HCSGM is a 24-item questionnaire assessing six dimensions: information, family inclusion, communication, technical skills, emotional needs, and overall satisfaction. After two independent forward translations and a back translation, a preliminary version was tested with caregivers of 10 patients who provided feedback using the thinking-aloud technique while completing the questionnaire. Interviews were analyzed using qualitative content analysis (QCA), leading to the final German version of the HCSGM.

**Results:**

The initial consensus meeting after the forward translations identified five areas for improvement. The backward translation was approved with no further changes. QCA revealed ten major and nine minor issues, which resulted in ten modifications to item wordings, question-wording, verbal anchors of answer options, and layout during a second consensus meeting. Those modifications resulted in the final version also being approved by the original authors.

**Conclusion:**

The German version of the PedsQL HCSGM is now available to measure HCS, a crucial PROM for clinical research and patient management. While qualitative methods have been employed to ensure equivalence, further studies should evaluate psychometric properties.

**Supplementary Information:**

The online version contains supplementary material available at 10.1186/s12955-026-02492-1.

## Introduction

Patient-reported outcome measures (PROMs) and experience measures (PREMs) are essential for patient management and clinical research, as required by most Core Outcome Sets (COS) established by the Core Outcome Measures in Effectiveness Trials (COMET) [1]. PROMs and PREMs evaluate subjective perceptions, such as health-related quality of life (HRQOL) [2, 3]. PROMs provide critical insights into patient well-being, with HRQOL being vital for patient health [4, 5], which is consequently reflected in many COS [6–11]. Measuring HRQOL is also key for immediate patient management [12, 13] and healthcare research [14, 15]. Generally, PROMs are especially important in pediatric care, where effective communication and assessments can be particularly challenging [2, 16, 17].

Clinical symptoms alone can neither capture the illnesses´ nor the treatment’s impact on children’s well-being. Exploring children’s experiences supports shared decision-making, improves communication, and identifies individual care needs [2]. This knowledge also influences healthcare policies and resource allocation [2, 17–19]. Healthcare satisfaction (HCS) is a critical PROM that reflects care quality from the patient’s perspective [20]. Additionally, HCS impacts other PREMs and PROMs, particularly HRQOL assessments [20, 21]. Mental health is also linked to HCS [22–24]. Overall, HCS correlates positively with adherence to treatment guidelines [22, 25, 26]. Thus, a reliable tool to measure HCS is vital for evaluating healthcare quality and its broader effects.

Current validated German instruments lack a generic focus, which is essential for treating various diagnoses in pediatric surgery contexts [27–32]. Therefore, a generic HCS instrument is required. The Pediatric Quality of Life Inventory (PedsQL) Generic Module, commonly used internationally in pediatrics, has been adapted for Germany [16, 33]. Additionally, the PedsQL provides a validated generic Healthcare Satisfaction Generic Module (HCSGM) [34], which was developed through a literature review, focus groups, interviews, and extensive testing. However, this module has not yet been translated or culturally adapted for Germany. This study reports the translation and cultural adaptation of the PedsQL-HCSGM for the German context.

## Methods

### Measure

The PedsQL 3.0 HCSGM [34] is a 24-item proxy-reported instrument with six dimensions: information (five items), family inclusion (four items), communication (five items), technical skills (three items), emotional needs (four items), and overall satisfaction (three items). Responses are provided on a 5-point Likert scale (0="never satisfied” to 4="always satisfied”), with an option for “not applicable” (N/A) that eliminates the item from scoring. Scores are converted to a 0-100 scale (0 = 0, 1 = 25, 2 = 50, 3 = 75, 4 = 100). A score is calculated if at least 12 items are completed. Higher scores indicate greater satisfaction. The instrument is provided by Mapi Research Trust, Lyon, France [35].

### Study design

This study was designed as a methodological cross-sectional study focusing on the translation and linguistic validation of the questionnaire. Situated in a pediatric surgery center at a tertiary hospital, from September 2022 to June 2023, the research team comprised a senior pediatric surgery consultant (JS), an academic psychological psychotherapist (AZ), and medical researchers (AP, CHS). AP, a female fifth-year medical student who conducted interviews after training in cognitive interviewing techniques, which included role-plays and pilot tests. She was unknown to the participants before the interviews. Ten caregivers of children aged 0–17, treated as inpatients from February to March 2023, were recruited through convenience sampling. Caregivers were parents living permanently with the patients. Exclusion criteria included mental disabilities, cognitive or reading disorders, and non-German speakers. Participation was voluntary and required informed consent. Participants were informed that this study was part of AP’s Ph.D. thesis.

### Cultural adaptation

**Fig. 1:**
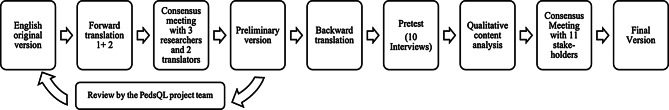
Schematic overview of the translation process

The local ethics committee approved the study (BO-EK-538122022). In a multi-step process, the translation and cross-cultural adaptation of the English-language questionnaire were conducted (Fig. 1). The translation process was performed as recommended [35], following guidelines for translating an instrument [36, 37] and enhanced with cognitive interviewing [38, 39] to achieve cultural equivalence [40–45]. Reporting adheres to the COnsolidated criteria for REporting Qualitative research (COREQ) [46] and the Standards for Reporting Qualitative Research (SRQR) [47] as suggested by the EQUATOR network [48].

*Phase 1 – Forward translation*: Two expert translators independently translated the PedsQL 3.0 HCSGM from English (source) to German (target). A native speaker ensured the accuracy of the translations through the four-eyes principle, verifying completeness, spelling, grammar, and proper term usage, adhering to DIN EN 15,038 and ISO 17,100. A consensus meeting involving the translators and three researchers combined the translations into a preliminary version, aiming for linguistic and formal equivalence.

*Phase 2 – Backward translation*: A certified bilingual translator converted the German version back into English for review by the PedsQL project team (San Diego, California, USA), directed by Dr. James Varni.

*Phase 3 – Pretest*: The translated questionnaire was presented to ten caregivers of pediatric surgical inpatients aged 0–17 during face-to-face interviews, with each participant being interviewed once. Cognitive debriefing utilized verbal probing and thinking-aloud techniques [49]. Participants expressed their thoughts and concerns while answering the questionnaires. After completing the preliminary German version of the PedsQL-HCSGM (G-HCSGM), semi-structured interviews with semi-open questions (supplementary 1) were conducted. Interviews were organized by questionnaire dimensions, with participants addressing one dimension at a time. Voice recordings captured participants thinking aloud during the questionnaires and interviews [38, 49]. Interviews took place in a quiet room at the clinic, sometimes with the child present.

### Qualitative Content Analysis (QCA)

After the interviews, AP and a research assistant transcribed audio recordings according to established rules [39]. The transcriptions were analyzed using MAXQDA software (Version 22.2.0; Germany), with supplementary audio tracks helping to capture voice tone and pitch, thereby detecting nuances such as sarcasm. Nonverbal communications noted during the interviews were added to MAXQDA’s memory function. The transcripts were not shared with participants. QCA was conducted by AP and AZ, who independently categorized the data using predefined categories based on the interviewer’s guidelines (concept-driven; supplementary 1). The initial coding adhered to predefined categories; however, a data-driven approach ultimately increased precision [38]. A consensus version emerged from both researchers’ codings with the revised system. Intercoder reliability was maintained as AP and AZ coded independently, leading to a combined coding version [39]. The coding process, including discussions and changes, was logged to enhance reliability, ensuring that any discrepancies led to more precise category definitions [50]. Issues raised by participants with the questionnaire and potential solutions were identified and discussed during a consensus meeting with 11 stakeholders. Interview participants were not included in the stakeholder meeting and therefore did not provide any feedback on the findings.

### Data analysis

Sociodemographic data were extracted from the electronic patient files using the hospital software ORBIS (Dedalus, DH Healthcare GmbH, Bonn, Germany) and evaluated with Microsoft Excel Version 16.84.

## Results

### Forward translations

Two independent forward translations were examined during a consensus meeting involving the two translators, JS, AZ, and a patient representative (mother of three children previously treated in the department). Five issues were identified: the title of the questionnaire, instructions (including answer options), the titles of the dimensions, general question wording, and item wording. Modifications were made for all five issues that were not included in either forward translation 1 or 2 (supplementary 2). A problem was identified where intensities were requested, but frequency-based answer options were provided; adjustments were made to ensure both reflected frequencies. The preliminary, unanimously consented version was sent for backward translation.

### Backward translation

The backward translation showed minimal differences, including the wording of the title, the instructions, the beginnings of the questions, and various phrasings of some items, compared to the source. These differences were considered minor. The MAPI Trust team and the instrument’s original author approved the backward translation without any changes. The preliminary forward translation was then tested with 10 participants.

#### Pretesting and cognitive interviewing

##### Participants’ and interview characteristics

The ten interviews lasted an average of 22.4 min (ranging from 14 to 42 min). The mean age of caregivers was 37.4 years (31 to 48), with an average of 2.2 children (2 to 3). The partners’ average age was 39.8 years (33 to 49). Caregivers’ children under medical treatment (patients) had an average age of 4.7 years (from 15 days to 17 years), with hospital stays averaging 5 days (3 to 8). One child had a care level of 2 out of 5, indicating a significant degree of dependence. Tables 1 and 2 present all sociodemographic data collected. Various characteristics were sensibly grouped into categories for comparability: e.g., “fracture with osteogenesis imperfecta and knee surgery” was termed “musculoskeletal disease”; “preschool” and other forms of daycare were grouped under “kindergarten.”

**Table 1:** Descriptive patient data (*n* = 10)

Age	Sex	Disease	Acute vs. chronic	Days in hospital	Surgery	Daycare	First visit to pediatric surgery	Care level
2 Y	Male	Musculoskeletal	Acute	5	Yes	Day nursery	Yes	0
6 Y	Female	Urogenital	Chronic	5	Yes	Kindergarten	No	0
2 Y	Female	Musculoskeletal	Chronic	7	Yes	Kindergarten	No	2
6 Y	Female	Visceral	Chronic	3	Yes	Kindergarten	No	0
1 W	Female	Visceral	Chronic	3	Yes	Care for at home	Yes	0
17 Y	Female	Visceral	Acute	7	Yes	School	No	0
5 Y	Female	Neurological	Acute	3	No	Kindergarten	Yes	0
35 W	Male	Neurological	Acute	8	No	Care for at home	Yes	0
8 Y	Female	Musculoskeletal	Acute	6	Yes	School	No	0
7 W	Male	Visceral	Acute	3	Yes	Care for at home	Yes	0

Under the German long−term care insurance system, care level 2 (Pflegegrad 2) indicates a moderate impairment of independence, corresponding to a defined need for assistance in daily activities

**Table 2:** Descriptive caregiver data (*n* = 10)

Care-giver	Age of caregiver	Occupation	Children	Maternal status	Partner	Age of Partner	Occupation partner
Mother	37	Non-Academic	2	Permanent relationship	Father	42	Non-Academic
Mother	48	Academic	2	Married	Father	49	Academic
Mother	41	Non-Academic	3	Married	Father	43	Non-Academic
Mother	35	Non-Academic	2	Married	Father	37	Non-Academic
Mother	35	Non-Academic	2 + 1 from other parent	Permanent relationship	Father	40	Non-Academic
Mother	(n. s.)	(n. s.)	2	Permanent relationship	Father	(n. s.)	(n. s.)
Mother	38	Academic	2	Married	Father	39	Academic
Mother	31	Non-Academic	2	Married	Father	37	Academic
Father	34	Non-Academic	2	Married	Mother	33	Non-Academic
Mother	38	Non-Academic	3	Married	Father	39	Non-Academic

Note: (n.s.) = (not specified). Classification of parents into academic and non−academic categories based on their occupations. The categorization was conducted according to whether the respective profession in Germany typically requires completion of a university degree in order to be practiced

### Qualitative content analysis

**Fig. 2:**
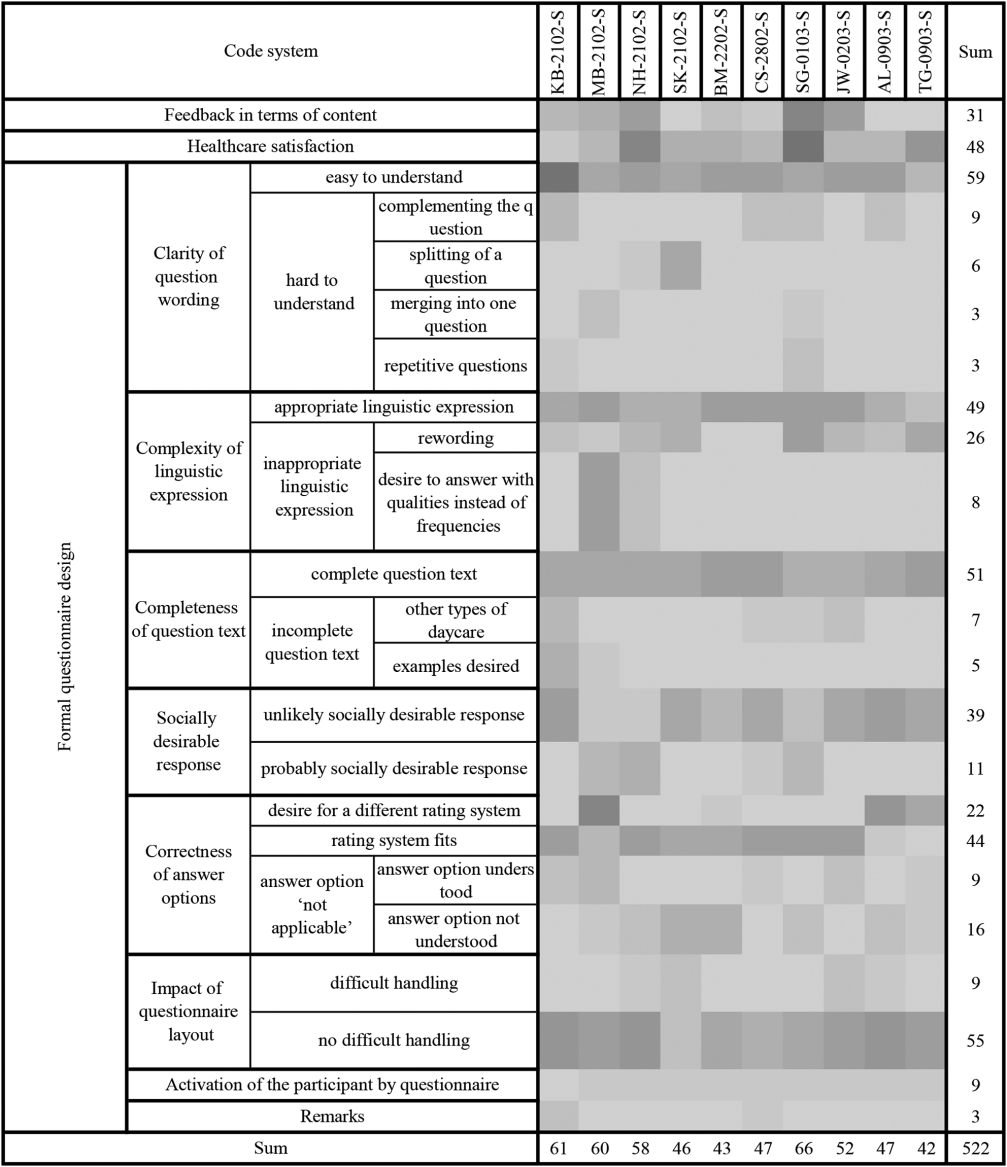
Heatmap illustrating the frequency of coded segments across interviews. Individual statements are grouped for each participant (columns) and assigned to coded categories (lines). Darker shading indicates higher coding frequency. The sum of the given codes can be evaluated separately for each code and participant. In total, 522 statements were coded

For QCA, the concept-driven approach revealed two domains: healthcare satisfaction, with two subscales, and formal questionnaire design, with eight subscales. The data-driven analysis identified an additional domain, “content-related feedback,” composed of eight subscales. It also refined the concept-driven subscales by modifying sub-subscales and eliminating two original subscales (Table 3). The assigned codes are displayed per participant (Fig. 2).

**Table 3:** Coding system with the english translations of the German codes

English Translation	Approach
*Formal questionnaire design*	C
Clarity of question wording	C
- easy to understand - hard to understand o complementing the question o splitting of a question o merging into one question o repetitive questions	D
Complexity of linguistic expression	C
- appropriate linguistic expression - inappropriate linguistic expression o rewording o desire to answer with qualities instead of frequencies	D
Completeness of question text	C
- complete question text - incomplete question text o other types of daycare o examples desired	D
Socially desirable response	C
- risk of dishonest answers - risk of non-response	CL
- unlikely socially desirable response - probably socially desirable response	D
Correctness of answer options	C
- desire for a different rating system - rating system fits - answer option ‘not applicable’ o answer option understood o answer option not understood	D
Impact of questionnaire layout	C
- difficult handling - no difficult handling	D
Activation of the participant by questionnaire	C
- interest in the questionnaire - attention to the questionnaire	CL
Remarks	C
*Healthcare satisfaction*	C
Suggestions for improvement of care in pediatric surgery	C
Resubmission if required to pediatrics surgery	C
Compliment	D
Critics	D
- More empathy - More time - Staff shortage - More feedback after surgery by the surgeon	D
*Feedback in terms of content*	D
Feedback on the content of the introduction	D
Feedback on the content of the whole questionnaire	D
Feedback to the content of section 1 (Information)	D
Feedback to the content of section 2 (Inclusion of family)	D
Feedback to the content of section 3 (Communication)	D
Feedback on the content of section 4 (Technical skills)	D
Feedback to the content of section 5 (Emotional needs)	D
Feedback to the content of section 6 (Overall satisfaction)	D

Approaches are concept-driven (C), data-driven (D), or concept-driven but do not fit the data and are therefore left out in the final coding system (CL). For the German codes related to the English translations see supplementary 3

This multi-step approach yielded key themes, which were presented during the 1.5-hour final consensus meeting with 11 stakeholders (Supplementary 4).

They ultimately translated into ten changes to the final version of the G-HCSGM:

*General question*: Four participants requested a different verbal anchor for the question, with two finding “frequently” to be inapplicable. The question was converted from “How often are you satisfied with …” (“Wie häufig sind Sie zufrieden mit …”) to “How satisfied are you with …” (“Wie zufrieden sind Sie mit …”).

*Not applicable*: Five participants did not understand the “not applicable” (“nicht anwendbar”) answer options, and two only partially understood it, which prompted a change to a more commonly used German translation (“nicht zutreffend”).

*Answer options*: Five participants reported issues with the scaling of the frequency-based answer options. One participant requested a change in the order of the answer options. Another wanted the number of answer options to be reduced. Additionally, one participant preferred school grades, and two participants generally noted a problem with the verbal anchors of the answer options being frequencies. In accordance with the general question, it was converted to intensities “not-little-moderately-quite-very much” (“nicht-wenig-mittelmäßig-ziemlich-sehr”).

*Layout of items*: Three pairs of items (3.1. / 3.2. and 3.4. / 3.5. and 5.3. / 5.4.) distinguish to whom the questions refer (child or adult). Four participants experienced difficulties with these questions. Consequently, the bolded words “to you” and “to your child” were underlined. One participant reported consistently needing to refer back to the instructions. Two participants noted issues related to the beginning of the question always being at the top of the page rather than directly in front of the item. These issues did not lead to changes to the G-HCSGM. One participant desired free-text answer options, but to maintain simplicity, this was not implemented.

*Socially desirable responses*: Four participants mentioned the risk of answering questions based on social norms to avoid offending anyone. This resulted in no change to the G-HCSGM, but it was agreed to distribute the G-HCSGM blinded at the end of the treatment.

*Wording issues*: One participant found the questionnaire’s name odd, but no change was made due to copyright constraints. Eight participants felt some wording may be complicated without specifying which. These comments led to the deletion of unnecessary word duplications, revising the sentence to: “Please answer [us] the following questions and tell us how satisfied you are with the care you, your child, and other family members received from hospital staff” (“Bitte beantworten Sie [für uns] die folgenden Fragen und teilen Sie uns mit, wie zufrieden Sie mit der Behandlung sind, die Sie, Ihr Kind sowie andere Familienmitglieder vom Krankenhauspersonal erhalten haben”). Question 2.4 had redundant staff references, reworded to: “… with time given to ask questions about your child’s health or treatment” (“… mit der Zeit, die Ihnen gegeben wurde, um Fragen zum Gesundheitszustand oder zur Behandlung Ihres Kindes zu stellen”). Five participants found questions 3.4 and 5 unclear regarding “preparation for examinations and treatments.” They were changed to: “… preparation for examinations and treatments of your child offered to your child by the staff” (“der Vorbereitung auf Untersuchungen und Behandlungen ihres Kindes, die Ihrem Kind durch das Personal angeboten wurde”) and “…preparation for examinations and treatments of your child offered to you by the staff” (“der Vorbereitung auf Untersuchungen und Behandlungen ihres Kindes, die Ihnen durch das Personal angeboten wurde”). One participant noted question 4.2 had overblown language, but no changes were made to the G-HCSGM. Another participant indicated “health condition” might not include an injury, yet it remains common terminology in German. Three participants found question 4.3 misleading, prompting a rewording to: “How satisfied are you with the staff’s efforts to prepare you for taking your child home?” (“Wie zufrieden sind Sie mit dem Bemühen des Personals, Sie bei der Rückkehr Ihres Kindes nach Hause zu unterstützen?“). Four participants pointed out that question 5.2 only mentioned school, leading to the change to “daycare facility/school” (“Tageseinrichtung/Schule”). Some participants requested more detailed questions, which were not incorporated into the final version, as most understood the questions without further elaboration. Two participants suggested new questions about the surgeon’s post-surgery visit; however, these were not incorporated, as the topics are already evaluated in the general information assessment. Two participants recommended eliminating certain questions or dimensions for emotional needs, which were deemed unnecessary. The stakeholder consensus meeting decided to maintain the original number of questions. All changes made during the consensus meeting were finally approved by the original authors.

## Discussion

Cross-cultural adaptation aims to ensure content consistency and face validity between the source and target versions of the questionnaire [36]. The first step is translating the source into the target language [36]. Literature reports various methods for this. One method is Chen and Boore´s multistep procedure, involving repeated forward and backward translations “until the target language is acceptably equivalent to the source language” [51]. Beaton et al. (2000) recommended one informed and one uninformed translator. The procedure chosen for this study combines multiple published methods as suggested by MAPI [35]. Following Beaton et al. (2000), we used two independent translators; however, contrary to their guidelines, neither was informed about the content and underlying concepts. Additionally, informed stakeholders, who verified content accuracy based on their in-depth knowledge as diverse healthcare professionals, were included in our consensus meeting on integrating both forward translations.

Despite using two certified native English translators and supervisory oversight, a consensus meeting on both forward translations revealed five issues. Consequently, the preliminary forward translation included wordings that were absent from either of the initial versions. This highlights the need for engaging stakeholders with expertise in medicine, psychology, nursing, and caregivers proficient in English. Only this approach can uncover problems that native speakers and expert translators might miss.

The next step was to evaluate the equivalence of the preliminary forward version to the source [36]. Backward translation is commonly used for this purpose, although it may not prove equivalence on its own [37, 52, 53]. Therefore, it can be combined with other techniques, such as pretesting [37]. Contrary to Beaton et al. (2000), we conducted pretesting before the stakeholder consensus meeting, as our back-translation closely matched the original and was approved without changes, including the approval by the original authors for the questions being initially changed from intensities: “How happy are you with … " to frequencies: “How often are you satisfied with …” to fit frequency-based answer options. This way, our second stakeholder consensus meeting was based on the pretest’s results and was thus more profitable.

To further test equivalence, we pretested with ten participants using cognitive interviewing and QCA. This procedure is essential, as cross-cultural comparisons of translations have shown that translators often accept the target measure on the basis of unjustified assertions about cultural applicability [40]. Furthermore, we concur with the previously mentioned study that “quantitative analysis is mainly the preferred approach, even when qualitative procedures would certainly prove to be equally effective” [40]. Additionally, these qualitative methods may yield more detailed findings than quantitative approaches.

Literature suggests varying participant numbers. Several issues influenced the discussion regarding sample size: after involving the fourth participant, no new issues were detected by the subsequent participants (Supplementary 5). Experimental saturation literature suggests that twelve participants can identify over 90% of codes found in much larger samples [54]. In our case, there were no new changes to the G-HCSGM from the fourth participant onward, leading us to conclude that ten participants were adequate, especially in light of resource efficiency. Moreover, none of the participants raised the concerns identified during the stakeholder consensus meeting, which resulted in modifications to the final version (items 6.1 & 6.2), underscoring the value of diverse stakeholder input over merely increasing participant numbers.

For QCA, issue frequency is less important than consensus. Even if only one participant noted missing types of daycare facilities, the issue would still be addressed, as stakeholders believed the absence of these facilities did not reflect the German context. Conversely, despite multiple mentions of “health condition” being an inappropriate term for injuries, it was retained. It did not change the G-HCSGM, as it was considered a common German term for disease, including injuries. Moreover, the QCA revealed that experts had transitioned from using questions of intensity to frequency as a problem. Contrary to the initial change, a conversion from frequencies (“How often are you satisfied with …” and answer options: Never-sometimes-often-almost always-always) to intensities (“How satisfied are you with …” and answer options: not-little-moderately-quite-very much) was executed and approved by the original authors. Mixing questions that measure intensity with frequency-based answer options poses a significant problem, as intensity gauges the strength of a feeling or belief. In contrast, frequency measures the number of times an event occurs. These two constructs are inherently incompatible, making it difficult to compare them directly. Nevertheless, shifting between frequencies and intensities would reduce comparability between the original English and German versions. The English HCSGM itself contains this inconsistency between questions and answers that our translation resolves. Finally, the consensus meeting was crucial for interpreting findings, as most participants identified problems without suggesting solutions, necessitating stakeholders to find and evaluate solutions.

Following cognitive interviews, ten improvements were implemented to optimize understanding and cultural relevance for German users. Most of these issues were identified during pretesting and the interviews. However, each translation step resulted in modifications, underscoring the importance of conducting each step rigorously and attentively. When the lack of detailed reporting in many prior qualitative studies was criticized [55], this study documents each step and its impact, providing transparency for future researchers assessing the G-HCSGM.

### Limitations

Sick children often wanted to stay with their caregivers, which occasionally led to distractions during interviews. However, interviews were paused as needed to minimize disruptions. Afterward, we provided a brief overview of the thinking-aloud technique, and the interview resumed after briefly revisiting the previous topic. Additionally, caregivers sometimes needed reminders that nonverbal communication was not audio-recorded; however, it was documented and could be utilized in the QCA. Others struggled with the “thinking-aloud” technique, answering questions directly instead of verbalizing their thoughts. Nevertheless, the structured interview addressed the most common issues arising from translations, and during the final questions, caregivers could share anything they had forgotten. The convenience sampling of participants may also represent another limitation. AP randomly invited inpatient caregivers to participate, estimating an inclusion rate of 50–60%, although exact figures were not recorded. However, based on the descriptive data, participants represented a diverse range of pediatric surgery patients (e.g., in terms of age and disease group). The caregivers also exhibited a wide range of ages, education levels, and occupations. One potential bias was the overrepresentation of mothers, who are more likely to stay in the hospital and agree to participate. Nonetheless, the sample still reflected broad variability. The stakeholders in the second consensus meeting were primarily PhD students, representing a specific perspective. However, due to their occupations and medical studies in Germany, which involve regular patient interactions, they are also engaged in patient care. Moreover, half of the stakeholders are caregivers for children themselves. According to Stevelink and van Brake (2013), cultural equivalence is multidimensional and includes semantic, operational, conceptual, theoretical, measurement, and item equivalence. This study addressed all aspects except theoretical and measurement equivalence, which will be evaluated in a future validation of the G-HCSGM.

## Conclusion

The PedsQL HCSGM was successfully translated and adapted to the German context, facilitating the assessment of HCS for clinical research and immediate patient management in pediatric populations. Subsequent studies are needed to evaluate the psychometric characteristics.

Declarations.

## Supplementary Information

Below is the link to the electronic supplementary material.

Supplementary Material 1

Supplementary Material 2

Supplementary Material 3

Supplementary Material 4

Supplementary Material 5

## Data Availability

The datasets used and analyzed in this study include interview recordings and transcripts as well as individual patient data that cannot be shared for data protection reasons.

## Abbreviations

COMET: Core Outcome Measures in Effectiveness Trials
COREQ: COnsolidated criteria for REporting Qualitative research
COS: Core Outcome Sets
DISQOL: DIsease-Specific Quality Of Life
G-HCSGM: German version of the PedsQL HealthCare Satisfaction Generic Module
HCS: HealthCare Satisfaction
HCSGM: the PedsQL HealthCare Satisfaction Generic Module
HRQOL: Health-Related Quality Of Life
PedsQL: Pediatric Quality of Life Inventory
PREM: Patient-Reported Experience Measure
PROM: Patient-Reported Outcome Measure
QCA: Qualitative Content Analysis
SRQR: Standards for Reporting Qualitative Research
