# Supplementary material for: German translation, cultural adaptation and linguistic validation of the PedsQL healthcare satisfaction module
Source: Health Qual Life Outcomes. 2026 Feb 13;24:28. doi: 10.1186/s12955-026-02492-1 (PMC12955180; doi:10.1186/s12955-026-02492-1)
Supplement: Supplementary file 1 — Supplementary Material 1 [file 12955_2026_2492_MOESM1_ESM.docx]

Supplementary 1: Interview guide: German questions and their translations.

| **German Question** | **Translation** |
| --- | --- |
| Enthält der Fragetext Worte/Fachausdrücke/ Formulierungen mit vager/unklarer Bedeutung? | Are there any words/technical terms/linguistic expressions with an unclear/vague meaning? |
| Kann der sprachliche Ausdruck vereinfacht werden? | Could the linguistic expression be simplified? |
| Ist der Fragetext unvollständig? | Is the question text incomplete? |
| Besteht die Gefahr, dass Fragen gar nicht oder nicht ehrlich beantwortet werden? | Is there a risk of not answering or answering a question dishonestly? |
| Gibt es Antwortvorgaben, die nicht zur Frage passen? | Are there possible answers that do not fit the question? |
| Erschwert die Gestaltung des Fragebogens die Bearbeitung? | Does the layout of the questionnaire impede the handling? |
| Wie schätzen Sie ihr Interesse und ihre Aufmerksamkeit am gesamten Fragebogen ein? | How do you evaluate your interest and your attention to the questionnaire? |
| Sonstige Anmerkungen | Any Additions? |
| Was könnte ihrer Meinung nach an der stationären Betreuung in der KCH verbessert werden? | In your opinion, what could be improved in inpatient care at pediatric surgery? |
| Würden Sie sich, wenn erforderlich, erneut  in die KCH am UKD in Behandlung begeben? | Would you consider being treated again at pediatric surgery in this hospital if necessary? |
