# Supplementary material for: German translation, cultural adaptation and linguistic validation of the PedsQL healthcare satisfaction module
Source: Health Qual Life Outcomes. 2026 Feb 13;24:28. doi: 10.1186/s12955-026-02492-1 (PMC12955180; doi:10.1186/s12955-026-02492-1)
Supplement: Supplementary file 2 — Supplementary Material 2 [file 12955_2026_2492_MOESM2_ESM.docx]

Supplementary 2: Results of the forward translation process.

| **Theme** | **Original** | **Forward Translation Version A** | **Forward Translation Version B** | **Consensus Forward Translation Version-AB** | **Explanation** |
| --- | --- | --- | --- | --- | --- |
| *Questionnaire title* | Healthcare Satisfaction  Generic Module  Parent Report | Generisches Modul zur Zufriedenheit im Gesundheitswesen  Elternbericht | Generisches Modul zur Zufriedenheit mit der Gesundheitsfürsorge  Elternbericht | Zufriedenheit mit der Gesundheitsversorgung  Generisches Modul  Fragebogen für Eltern | Version A implies that the satisfaction of health care workers could be meant, and version B means how regularly one perceives, e.g., preventive medical check-ups: The original content clearly describes aspects that relate to the individually received health care at that instance, which best translates in the German-speaking world to the consensus version. |
| *Instruction (including scale)* | Never,  sometimes,  often,  almost always,  always | überhaupt nicht, manchmal,  oft,  meistens,  immer | nie,  manchmal,  oft,  meistens,  immer | nie,  manchmal,  oft,  meistens,  immer | Scale division based on forward version B (shorter, more transparent, and based on typical Likert scale in German).  Further text: Mainly based on forward version A, simplification of the wording to improve comprehensibility for the users. |
| *Dimensions*  *(e.g. information)* |  | Rubrik: Informationen | Rubrik:  Informationen | Rubrik:  Informationen | The translator's proposal (forward version A) was discarded, and agreement was reached on the simple name of the dimension, "Information." |
| *General question* | How happy are you with… | Wie zufrieden sind Sie mit: | Wie zufrieden sind Sie mit: | Wie häufig sind Sie zufrieden mit: | Consensus versions A and B use an introductory question about quality, whereas the scale division asks about quantity/frequency—agreement on adding "frequently" as quantitative terminology. Otherwise, the answers "never, sometimes, often, almost always, and always do not fit with the questions. |
| *Items (e.g. item 1)* | … How much information was provided to you about your child’s diagnosis | … Wie viele Informationen Sie über die Diagnose Ihres Kindes erhalten haben | … Der Menge an Informationen, die Sie über die Diagnose Ihres Kindes erhielten | … Den Informationen, die Sie über die Diagnose Ihres Kindes erhalten haben | Adaptation of the items to the changed general initial question and joint agreement on one version each and subsequent simplification of the wording |
