# Supplementary material for: German translation, cultural adaptation and linguistic validation of the PedsQL healthcare satisfaction module
Source: Health Qual Life Outcomes. 2026 Feb 13;24:28. doi: 10.1186/s12955-026-02492-1 (PMC12955180; doi:10.1186/s12955-026-02492-1)
Supplement: Supplementary file 3 — Supplementary Material 3 [file 12955_2026_2492_MOESM3_ESM.docx]

Supplementary 3: Coding system with German codes and their translations

| **German Code** | **English Translation** | **Approach** |
| --- | --- | --- |
| *Formale Fragebogengestaltung* | *Formal questionnaire design* | C |
| Klarheit Frageformulierung | Clarity of question wording | C |
| - Gut verständlich - Schwer verständlich   - Ergänzung der Frage   - Trennung einer Frage   - Zusammenfassen zu einer Frage   - Wiederholung | - easy to understand - hard to understand   - complementing the question   - splitting of a question   - merging into one question   - repetitive questions | D |
| Schweregrad sprachlicher Ausdruck | Complexity of linguistic expression | C |
| - Angemessener sprachlicher Ausdruck - Unangemessener sprachlicher Ausdruck   - Umformulieren   - Wertigkeit statt Häufigkeit gewünscht | - appropriate linguistic expression - inappropriate linguistic expression   - rewording   - desire to answer with qualities instead of frequencies | D |
| Vollständigkeit Fragetext | Completeness of question text | C |
| - Vollständiger Fragetext - Unvollständiger Fragetext   - Tageseinrichtung   - Beispiele erwünscht | - complete question text - incomplete question text   - other types of daycare   - examples desired | D |
| Sozial erwünschte Beantwortung | Socially desirable response | C |
| - Gefahr unehrlicher Beantwortung - Gefahr fehlender Beantwortung | - risk of dishonest answers - risk of non-response | CL |
| - Sozial erwünschte Beantwortung unwahrscheinlich - Sozial erwünschte Beantwortung wahrscheinlich | - unlikely socially desirable response - probably socially desirable response | D |
| Korrektheit Antwortmöglichkeiten | Correctness of answer options | C |
| - anderes Bewertungssystem erwünscht - Bewertungssystem passt - Antwortmöglichkeit „nicht anwendbar“   - Antwortmöglichkeit verstanden   - Antwortmöglichkeit nicht verstanden | - desire for a different rating system - rating system fits - answer option ‘not applicable’   - answer option understood   - answer option not understood | D |
| Einfluss Fragebogenlayout | Impact of questionnaire layout | C |
| - Erschwerte Bearbeitung - Keine erschwerte Bearbeitung | - difficult handling - no difficult handling | D |
| Aktivierung des Ausfüllenden durch Fragebogen | Activation of the participant by questionnaire | C |
| - Interesse am Fragebogen - Aufmerksamkeit am Fragebogen | - interest in the questionnaire - attention to the questionnaire | CL |
| Anmerkungen | Remarks | C |
| *Behandlungszufriedenheit* | *Healthcare satisfaction* | C |
| Verbesserungsvorschläge  Betreuung in der KCH | Suggestions for improvement  of care in pediatric surgery | C |
| Wiedervorstellung KCH | Resubmission if required to pediatrics surgery | C |
| Lob | Compliment | D |
| Kritik | Critics | D |
| - Mehr Empathie - Mehr Zeit - Personalmangel - Rückmeldung nach OP von Operateur | - More empathy - More time - Staff shortage - More feedback after surgery  by the surgeon | D |
| *Inhaltliche Rückmeldungen* | *Feedback in terms of content* | D |
| Inhaltliche Rückmeldung zur Einleitung | Feedback on the content of the introduction | D |
| Inhaltliche Rückmeldung zum Gesamtfragebogen | Feedback on the content of the whole questionnaire | D |
| Inhaltliche Rückmeldung zu Abschnitt 1 (Informationen) | Feedback to the content of section 1  (Information) | D |
| Inhaltliche Rückmeldung zu Abschnitt 2 (Einbeziehung der Familie) | Feedback to the content of section 2  (Inclusion of family) | D |
| Inhaltliche Rückmeldung zu Abschnitt 3 (Kommunikation) | Feedback to the content of section 3  (Communication) | D |
| Inhaltliche Rückmeldung zu Abschnitt 4  (Fachliche Kompetenzen) | Feedback on the content of section 4  (Technical skills) | D |
| Inhaltliche Rückmeldung zu Abschnitt 5 (Emotionale Bedürfnisse) | Feedback to the content of section 5  (Emotional needs) | D |
| Inhaltliche Rückmeldung zu Abschnitt 6 (Zufriedenheit insgesamt) | Feedback to the content of section 6  (Overall satisfaction) | D |

^Approaches are concept-driven (C), data-driven (D), or concept-driven but do not fit the data and are therefore left out in the final coding system (CL).^
