# Supplementary material for: German translation, cultural adaptation and linguistic validation of the PedsQL healthcare satisfaction module
Source: Health Qual Life Outcomes. 2026 Feb 13;24:28. doi: 10.1186/s12955-026-02492-1 (PMC12955180; doi:10.1186/s12955-026-02492-1)
Supplement: Supplementary file 4 — Supplementary Material 4 [file 12955_2026_2492_MOESM4_ESM.docx]

Supplementary 4: Stakeholders participating in the consensus meeting (n=11).

| Stakeholder | Gender | Occupation | Children |
| --- | --- | --- | --- |
| CP | male | Research assistant, pediatric nurse, study nurse | --- |
| JS | male | Senior pediatric surgery consultant | 3 |
| AP | female | PhD student / medical researcher | 1 |
| CHS | female | PhD student / medical researcher | --- |
| JA | female | Patient representative | 3 |
| PE | female | PhD student / medical researcher, pediatric nurse | --- |
| HW | female | PhD student / researcher, physiotherapist | --- |
| DJ | male | PhD student / medical researcher | --- |
| HH | male | PhD student / medical researcher | --- |
| KH | male | Nurse, physician assistant | 2 |
| AZ | female | Academic psychological psychotherapist, nurse | 2 |
