# Supplementary material for: German translation, cultural adaptation and linguistic validation of the PedsQL healthcare satisfaction module
Source: Health Qual Life Outcomes. 2026 Feb 13;24:28. doi: 10.1186/s12955-026-02492-1 (PMC12955180; doi:10.1186/s12955-026-02492-1)
Supplement: Supplementary file 5 — Supplementary Material 5 [file 12955_2026_2492_MOESM5_ESM.docx]

Supplementary 5: Table of mentioned issues leading to a change of the G-HCSGM.

| **Issues** | KB-2102-S | MB-2102-S | NH-2102-S | SK-2102-S | BM-2202-S | CS-2802-S | SG-0103-S | JW-0203-S | AL-0903-S | TG-0903-S | Sum of participants mentioning this issues |
| --- | --- | --- | --- | --- | --- | --- | --- | --- | --- | --- | --- |
| 1. Wording of the questions |  | X  (X) | (X) |  | X |  |  |  | X | X | 4/10  (5/10) |
| 1. Wording „Not applicable“ |  | X | X | X | X |  | X |  | X | X | 7/10 |
| 1. Likert-Scale |  | X  (X) | (X) |  | X |  |  |  | X | X | 4/10  (5/10) |
| 1. Layout |  |  |  | X |  | X | X |  | X |  | 4/10 |
| 1. Socially desirable answers |  | X | X |  |  | X | X |  |  |  | 4/10 |
| 1. Wording |  |  |  |  |  |  |  |  |  |  |  |
| 6.1 Word duplications |  |  |  |  |  |  |  |  |  |  | 0/10 |
| - 1. Redundant references to staff |  |  |  |  |  |  |  |  |  |  | 0/10 |
| 6.3 Other types of daycare not mentioned | X |  |  |  |  | X | X | X |  |  | 4/10 |
| 6.4 To whom refers „preparation for examination and treament" |  |  |  | X |  | X | X | X | X |  | 5/10 |
| 6.5 What is „Assistance returing home“ |  | X | X |  |  |  |  | X |  |  | 3/10 |

The table illustrates which participants identified issues with the G-HCSGM that resulted in changes to the final version. It indicates that from the fourth participant onward, every issue that led to modifications of the G-HCSGM was mentioned at least once. An exception are the wording changes (6.1 & 6.2). These were based on unspecified concerns regarding the wording, such as when participants noted that something sounded strange. The consensus group made these changes without direct input from the participants.
